# Factors affecting youth empowerment and entrepreneurial initiatives: Social implications and way forward

**DOI:** 10.3389/fpsyg.2022.912259

**Published:** 2022-10-05

**Authors:** Anam Javeed, Mohammed Aljuaid, Sajid Mehmood, Muhammad Yar Khan, Zahid Mahmood, Duaa Shahid, Syed Sikandar Wali

**Affiliations:** ^1^Department of Management Sciences, University of Wah, Wah Cantt, Pakistan; ^2^Department of Health Administration, College of Business Administration, King Saud University, Riyadh, Saudi Arabia; ^3^Department of Management and Administrative Sciences, University of Gujrat, Gujrat, Pakistan; ^4^Department of Management Sciences, COMSATS University Islamabad, Wah Campus, Wah Cantt, Pakistan; ^5^Department of Management, College of Business Administration, King Saud University, Riyadh, Saudi Arabia; ^6^Hult International Business School, Cambridge, MA, United States

**Keywords:** entrepreneurial initiatives, social engagement, youth empowerment, government responsibility, lack of participation

## Abstract

This study aims to identify the factors that impact the empowerment of Pakistani youth and their entrepreneurial initiatives in Pakistan. A sample of 350 youngsters from renowned Pakistani universities across the country was selected for this purpose. The questionnaire was administered in person and electronically. Statistical Package of Social Sciences (SPSS) was used for analyzing the data. The findings indicated that all the hypothesized factors (government policies, lack of political participation, employment opportunities, and social engagement) had an impact on youth empowerment and their ability to initiate entrepreneurial activities. This study identified the set of factors that impact empowerment in youngsters, and this model can be extended to other contexts and additional factors can be included. The analytical findings from this study serve to help the government formulate appropriate policies for underserved youth and include them in the policy-making process so that the factors that hinder their empowerment and entrepreneurial initiatives could be addressed.

## Introduction

Entrepreneurship is related to the creation of new ventures and processes. The entrepreneurial initiatives taken by youngsters form the pillars of socio-economic development of the country (Kloepfer and Castrogiovanni, 2018; Widyanti and Rajiani, 2021). Young people who possess entrepreneurial intentions are likely to take initiative rather than rely on government employment opportunities (Liu et al., 2019). The factors that affect entrepreneurial initiatives have been studied by various researchers and analysts such as Gwija and Iwu (2014) in Saudi Africa, Almobaireek and Manolova (2013) in Saudi Arabia, Almobaireek and Manolova (2013) in Canada, and Widyanti and Rajiani (2021) in Indonesia.

A significant portion of the global population today is young. It is evident that the youth can bring about social and economic change (Hafeez and Fasih, 2018). Specifically, in developing nations, youth are a majority and can play a vital role in the development of the nation. To have productive national policies and foster economic development, engagement of youth is deemed necessary (A. Ali et al., 2017). Mobilizing and empowering the youth can help the nation to prosper. This study intends to explore the current level of unemployment among young people residing in Pakistan relative to their social, political, and economic issues (Holford, 2020), and also identify the challenges being faced by them with an aim to offer recommendations to policymakers.

Reduction of unemployment among the youth has become one of the most difficult challenges of the current era. According to ILO (2005), a reduction in youth unemployment can significantly add to the country's GDP and accrue economic gains to society. By empowering youngsters, the rate of violence and crime can be decreased. It also provides young people a sense of belonging and opportunities to achieve their visions and dreams. Hence, relevant programs and policies focused on youth employment are required for the country to benefit all in the long run (ILO, 2005).

It is a sad reality that youth in the country are energetic and full of potential, but it has not been properly directed and channelized. It is undeniable that youth have a role to play in policy making and national development, but they have been kept away from national policies which hits their self-esteem (Long et al., 2021). The consequence of it is reflected in the ongoing multitude of challenges the country is confronted with, including negative processes of sociocultural change such as extremism and intolerance, impoverished political and democratic culture, and a dwindling economy. The level of youth engagement in Pakistan is quite low compared to the youth demography in the country. The responsibility on the part of the government is wanting. The government and administration in the country should accept their social responsibility in encouraging entrepreneurial activities that provide youth with ideas and interest-free loans, organize awareness campaigns and seminars, and create opportunities for self-empowerment.

Various annual reports estimate youth to constitute about 30% of Pakistan's population. According to the United Nations Medium Variant Projections, Pakistan will reach its peak youth population in 2039 with an annual growth rate of 0.9% from 2025 to 2035. Although youth around the world face various challenges, the issues that Pakistani youth face are primarily regarding empowerment and entrepreneurial initiatives It has been established by Ali et al. (2012) that basic education, health, extremism, unemployment, and lack of political participation are the big issues which Pakistani people are facing. The involvement of young people to address the root causes of some of these issues are at a minimum. In, Similarly, the representation of youth in the political system of Pakistan is a big challenge and if they are well-represented then there can a good reflection of youth-related issues in the political sphere. Young people are the representatives of a nation, but in Pakistan, the youth seem to be helpless and directionless. The sustainable development goals (SDGs), particularly SDG 8 and SDG 10 focus on decent work and economic growth and addressing inequality. Pakistan is lagging in achieving many of the SDGs, but particularly relating to the employment targets, and the level of achievement is abysmal.

Pakistan is fortunate to have a young demographic profile that will remain high for another decade. It has been estimated that the population of people below the age of 30 is ~68%, hence the empowerment of such a big segment of the population will be a big investment for Pakistan's future (Rizvi and Nishat, 2009). Although the youth population is quite high in number yet development indicators show that around 30% of the population are illiterate and 77% of them could not complete their studies (UNDP, 2020).

Among the labor class in Pakistan, young people comprise 36.9% of the population, but their share as entrepreneurs is very less. High illiteracy, low education, lack of skills training, and scarce facilities and resources are some of the specific challenges young people encounter in the country. Every year four million Pakistani youth are added to the labor force whereas job creation caters to just one million hence creating immense frustration among young people (UNDP, 2020). Additionally, COVID-19 has intensified these pre-existing challenges and pressures further. Apart from the social and economic burdens on the country, the pandemic has had an unparalleled impact on young people. As of April 2020, Pakistan was one among many countries where education was switched to online mode, which has further created time and opportunities for online entrepreneurial activities. Youth, being the largest segment of the country, can make significant contributions to society through entrepreneurial activities. They can use social media and other digital platforms to spread positivity and eliminate poverty. Their initiatives can be expanded and replicated at the macro level engaging many people to benefit the masses. The youth can also actively participate as workers, managers, and entrepreneurs to support economic recovery, by innovating and finding solutions to the problems arising from the ongoing crisis, through the creation of new products and services using technology and other platforms.

The main objective of the study is to assess which factor among the selected factors of lack of political participation, government policies, employment opportunities, and social engagement have the most impact on empowerment and entrepreneurial initiatives. Data for the study was collected from the provincial and federal capitals of the country through in-person administration of the questionnaire as well as digital administration through G-form.

## Literature review

The empowerment of young people is an important factor that encourages youngsters to participate in the country's decision-making processes. Their inclusion in all kinds of policy-making and approval procedures is necessary. Empowerment can occur both at individual and collective levels and scholars such as Aquino et al. (2018) and Haldane et al. (2019) have considered empowerment as a tool that not only ushers changes at a personal level but also at collective, political, and societal levels. The reasons behind the difficult time Pakistani youth are facing is due to uncertain and adverse economic conditions and they are not being left with much of employment opportunities. This reality makes them least interested in making any effort toward the prosperity and development of the country. The majority of youngsters aim to leave the country and move abroad (Khalid and Asad, 2019).

Fayolle and Liñán (2014) call for studying the empowerment and entrepreneurial intentions of youth in various contexts to increase the level of their participation. It has further been emphasized (Thompson, 2009) that there exists a need to study the determining factors which hinder entrepreneurial intentions of youth and weaken their level of motivation. The attitude toward entrepreneurial intentions is the state of mind which motivates an individual to carry out developmental activities (Akanbi and Owoseni, 1988; Mahmoud and Garba, 2019). Discussing the Pakistani youth-related issues, Lall (2014) emphasizes that youngsters demand change in the political leadership of the country. It has been observed that all the political parties claim youth participation however in reality the level of their participation is unexpectedly low. According to Babbar and Qazilbash, 2004), youth unemployment and development issues are directly proportional to stress and social comparisons. Such conditions call for immediate response in the form of youth entrepreneurial and development policies. Unskilled and unemployed youth constitute a large proportion of the Pakistani population. Due to the poor economic conditions of their families, most young people are compelled to enter the job market without relevant skills hence increasing the percentage of unskilled workers in the country (Zia and Rehman, 2011).

Despite these challenging conditions, Pakistani youth are energetic and willing to improve their lives (Sadaf, 2012). The main responsibility remains with the government for designing policies that create opportunities for the youth to engage in entrepreneurial activities. Youth at every level, either urban or rural, are facing challenges. Young men and women experience numerous hurdles at every level. The reality is that government agencies continue to give hope and promises which are never actualized. The private sector too should be playing its part in the development of the young generation. The lack of motivation in being entrepreneurial is also due to the lack of youth representation in politics. The image of the political parties in the country is unattractive due to their track record. Integrating the youth into the political mainstream is essential for regaining their trust and will be a big task for any government (Rumi and Nauman, 2013).

According to recent demographic projections, Pakistan is one of the few countries with a large youth bulge. Given their huge numbers, the bottled-up energies and untapped potential of the youth put a big stress on the government. The government of Pakistan by itself is not capable of handling the demands of employment and empowerment. This article proposes that public and private joint ventures can help to empower youth, employ them, utilize their capabilities, and eventually contribute to the economic growth of the country and the development of its society.

In the context of a fragile democracy like Pakistan where the political system is constantly under stress, the involvement of youth is challenging. Although the youth are a majority in the population, they tend to be helpless, aimless, and powerless (Jiang, 2021). Limited youth participation in politics is an indication of their lack of confidence in the government's policies. Policies require to be well-structured, especially if they aim to revive confidence among youth and bring them into the economic mainstream of the country. According to Ahmad and Azim (2010), the low involvement of youth in the political system along with minimal government investment in youth development can be a threat to the health of the society. The lack of youth involvement, empowerment, and employment was a major concern in the millennium development goals (MDGs) and continues to be so in the SDGs as well.

## Conceptual framework and hypotheses development

### Governmental policies

Entrepreneurial activities are necessary for society and the healthy economy of the nation. Entrepreneurial activities carried out by small and medium enterprises create employment prospects for youngsters. Entrepreneurial activity is the combined effect of risk-taking and innovation. Entrepreneurial activities can further lead to the development of the country, particularly a developing country, where the value of entrepreneurial activities is greater compared to other developed economies. Unfortunately, entrepreneurial activities are limited in Pakistan. Further, the government is also not seen promoting these activities. It is obvious that the government does not take initiative to create entrepreneurial opportunities for youth and the private sector is expected to step in for motivating the youth. It has been noted by Haque (2007) that to enhance the entrepreneurial activities in the country, the government needs to relook its policies regarding rent, inspire novelty, and develop a culture favorable to business development. Formation of business-friendly strategies and empowerment of youth are the basic steps which are needed to be taken. Furthermore, in the role of the government in the establishment of small and medium-sized enterprises studied by Chemin (2009), it was established that youth employment, taxes, rents, etc. are the hurdles that a government should consider easing for encouraging entrepreneurial initiatives in Pakistan. It can be hypothesized that

**H**_**1**_**: Government policies have an impact on youth empowerment and entrepreneurial initiatives**.

### Lack of political participation

Pakistani youth are under stress which is an indicator of the problem in society. Despite their potential, the youth have been underutilized and unskilled despite spiraling unemployment rates. To address this situation, various initiatives like the Prime Minister's youth program and educational reforms have been proposed by every government, but ironically, they have failed to achieve the projected results. According to the Pakistan Labor Survey Report (2018–19), the number of people seeking jobs has increased. Since the youth do not see the government promises being kept, their political participation is also less. Without youth being involved in the political process of Pakistan, youth-focused governmental strategies are unlikely to be developed. Youth development measures and initiatives for empowering them have fundamentally not been very successful. Against this background, the government must undertake a gap analysis of the current youth condition and the shortcomings in the policies targeting youth which can ultimately alleviate the level of depression and stress among youth. It can be hypothesized that the lack of youth political participation has an impact on youth empowerment and entrepreneurial initiatives.

**H**_**2**_**: Lack of youth political participation has an impact on youth empowerment and entrepreneurial initiatives**.

### Employment opportunities

The current situation of unemployment in Pakistan is due to the adverse economic and political conditions prevailing in the country. Despite being a country with countless resources, because of political instability, the economic growth of the country has suffered significantly. The political situation of the country has an enormous impact on the economic well-being of the country. Unemployment impacts youth and has adverse societal impacts (Taha et al., 2016). Unemployed youth get caught in a cycle of dissatisfaction and depression. This also leads to unrest in society and negative developments such as street crimes. It has been emphasized by Lucifora and Moriconi (2015) that the political situation obtaining in a country attracts foreign direct investment. The unstable political situation in Pakistan, therefore, impacts domestic and foreign investment patterns.

**H**_**3**_**: Lack of employment opportunities has an impact on youth empowerment and entrepreneurial initiatives**.

### Social engagement

The social engagement of young people in the business sector of the country can enhance the economic conditions of the country. Business leaders can help youngsters who are facing resource constraints or lack motivation. In today's globalized world, there is an ever-emerging need for various types of entrepreneurial products and services. Social engagement can result in social entrepreneurship which increases the social and economic capital of the country and also encourages the youth toward entrepreneurial activities (Kraus et al., 2014) eventually contributing to societal well-being (Shaw and Carter, 2007; Dacin et al., 2010). To get insights from Pakistan, it is hypothesized that

**H**_**4**_**: Social engagement of youth has an impact on youth empowerment and entrepreneurial initiatives**.

**Figure 1:**
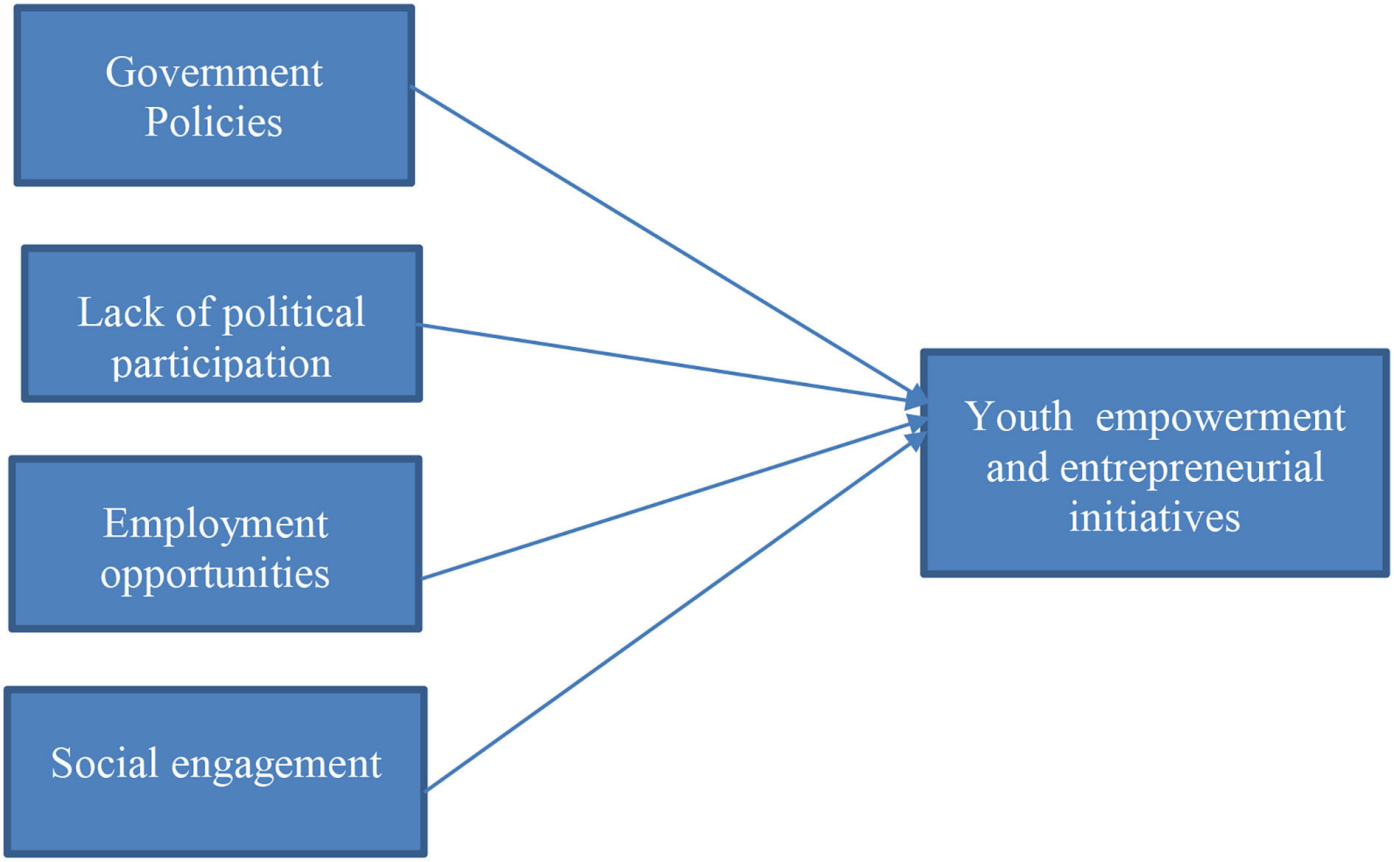
Conceptual framework.

The Figure 1 shown below exhibits the conceptual framework of the variables under study.

## Methodology

### Research design

This study was carried out with a positive paradigm. The main purpose of this study is to test the hypothesized relationships within the proposed conceptual framework. The study adopted a cross-sectional design. The gathered data were analyzed using the Statistical Package for the Social Sciences (SPSS). The data was collected from youth from provincial and federal capitals of Pakistan to ensure generalizability. After analyzing data from individual cities, a comparative analysis was carried out between the results from different cities. The questionnaire adapted from Fletcher (2015), Soomro and Shukui (2015); and Vethirajan et al. (2019) has been used as a tool for data collection through in-person distribution and via email. A stratified random sampling technique has been applied in the study because of geographical data collection. The capital cities have been considered as strata and the results were compared further on in the study.

### Model summary

The data gathered from the samples was put to analysis in SPSS. The results show satisfactory outputs. In the model summary shown in Table 1 the value of R shows a 45.5% correlation between the independent and dependent variables. However, from Table 1 it is evident that 19.1% of the variance in the dependent variable is caused by the independent variable. All the results are within the acceptable range.

**Table 1:** Model summary.

**Model**	**R**	**R squared**	**Adjusted R squared**	**Std error estimation**
1.	0.4550	0.207	0.191	0.4595

For ranking the factors that impact the entrepreneurial initiatives of youngsters, the questionnaire used the Likert scale. The data collection involved gathering details from all the big cities of Pakistan. Table 2 shows the details of the cities.

**Table 2:** List of selected universities.

**Cities**	**Universities**
Islamabad	• COMSATS University Islamabad • International Islamic University Islamabad
Lahore	• The university of Lahore • University of Central Punjab
Karachi	• Federal Urdu University Karachi • SZABIST Karachi
Quetta	• University of Baluchistan • Baluchistan University of Information Technology, Engineering and Management Sciences
Peshawar	• Abasyn University • Islamia College University

A sample of 1,750 respondents was selected to generalize the findings of the study. The questionnaire was circulated both manually and through online modes. Youth from selected universities all over Pakistan were selected as respondents, as the study is focused on their demography. The methodological basis of the study was quantitative in which the questionnaire was used to gather the data. A sample of 500 respondents was taken from each of the universities.

## Analysis

To analyze the data collected from the questionnaires, SPSS was applied. The software is widely used in social sciences studies. The basic information regarding the cities of Pakistan is shown in the tables below.

### Islamabad

Islamabad is the capital city of Pakistan which has the highest number of amenities/resources when compared to the rest of the country. The capital city is also representative of residents from other parts of the country. The results from Islamabad are shown in Table 3. The results indicate that youth from Islamabad consider the policies devised by the government to impact youth empowerment and employment (t = 12.104; *p* = 0.000). The creation of job opportunities is one of the prime responsibilities of the government and if they are unable to do so, the youth are distressed. The policies of the government on empowering the youth of the country act as a motivating factor. Due to the environment in the country where everyone waits for getting a good job, there exists a general lack of drive in the youngsters to come up with entrepreneurial ideas. The results show a lack of youth participation in entrepreneurial activities (t = 24.841; *p* = 0.000). The lack of adequate employment opportunities due to adverse economic conditions also has a direct impact on youth empowerment and entrepreneurship (t = 12.161; *p* = 0.000). The results reveal that the social engagement of youth in different societal activities promotes their entrepreneurial activities and empowerment.

**Table 3:** Regression analysis (Islamabad).

**Sr. No**	**Relationship**	**T-Value**	***P*** **-Value**	**Decision**
1.	Government policies → youth empowerment and entrepreneurial initiatives	12.104	0.000	Accepted
2.	Lack of youth participation → youth empowerment and entrepreneurial initiatives	24.841	0.000	Accepted
3.	Employment opportunities → youth empowerment and entrepreneurial initiatives	12.161	0.000	Accepted
4.	Social engagement → youth empowerment and entrepreneurial initiatives	4.581	0.000	Accepted

GP, Government policies; LYP, Lack of youth participation; EO, Employment opportunities; SE, Social engagement; YEI, Youth empowerment and employment initiatives.

### Karachi

Karachi is the biggest and most populated city in Pakistan. It is also the city that offers the maximum number of opportunities. Youth from Karachi consider that policies made by the government on every level directly or indirectly impact their empowerment and employment (t = 14.113; *p* = 0.000). Lack of youth participation (t = 3.225; *p* = 0.014) and social engagement (t = 3.001; *p* = 0.012) were not as influential as other factors. Employment opportunities also demonstrated an impact on youth employment and empowerment. The results are shown in Table 4.

**Table 4:** Regression analysis (Karachi).

**Sr. No**	**Relationship**	**T-Value**	***P*** **-Value**	**Decision**
1.	Government policies → youth empowerment and entrepreneurial initiatives	14.113	0.000	Accepted
2.	Lack of youth participation → youth empowerment and entrepreneurial initiatives	3.255	0.013	Accepted with minor effect
3.	Employment opportunities → youth empowerment and entrepreneurial initiatives	13.223	0.000	Accepted
4.	Social engagement → youth empowerment and entrepreneurial initiatives	3.001	0.012	Accepted with minor effect

GP, Government policies; LYP, Lack of youth participation; EO, Employment opportunities; SE, Social engagement; YEI, Youth empowerment and employment initiatives.

### Lahore

Lahore is one of the historical cities in the country with a huge population. Youth from Lahore felt that all the identified factors of poor government policies, lack of youth participation, scarce employment opportunities, and lack of social engagement impacted youth empowerment. The results are shown in Table 5.

**Table 5:** Regression analysis (Lahore).

**Sr. No**	**Relationship**	**T-Value**	***P*** **-Value**	**Decision**
1.	Government policies → youth empowerment and entrepreneurial initiatives	13.227	0.000	Accepted
2.	Lack of youth participation → youth empowerment and entrepreneurial initiatives	12.255	0.000	Accepted
3.	Employment opportunities → youth empowerment and entrepreneurial initiatives	13.223	0.000	Accepted
4.	Social engagement → youth empowerment and entrepreneurial initiatives	11.001	0.001	Accepted

GP, Government policies; LYP, Lack of youth participation; EO, Employment opportunities; SE, Social engagement; YEI, Youth empowerment and employment initiatives.

### Peshawar

Peshawar, also one of the oldest cities in Pakistan is a cultural and economic hub of Khyber Pakhtunkhwa. The youth from Peshawar also considered all the identified factors to be impacting youth empowerment and entrepreneurship. The policies devised by the government (t = 10.227; *p* = 0.001), lack of youth participation (t = 12.674; *p* = 0.001), employment opportunities (t = 11.484), and lack of social engagement (t = 12.001; *p* = 0.000) all had an effect on the motivation of youngsters to engage in the entrepreneurial activities and their empowerment levels. Table 6 shows the evidence of the results.

**Table 6:** Regression analysis (Peshawar).

**Sr. No**	**Relationship**	**T-Value**	***P*** **-Value**	**Decision**
1.	Government policies → youth empowerment and entrepreneurial initiatives	10.227	0.001	Accepted
2.	Lack of youth participation → youth empowerment and entrepreneurial initiatives	12.674	0.000	Accepted
3.	Employment opportunities → youth empowerment and entrepreneurial initiatives	11.484	0.001	Accepted
4.	Social engagement → youth empowerment and entrepreneurial initiatives	12.001	0.000	Accepted

GP, Government policies; LYP, Lack of youth participation; EO, Employment opportunities; SE, Social engagement; YEI, Youth empowerment and employment initiatives.

### Quetta

Quetta is another big city in Pakistan where the number of unemployed youth is high. Similar to other cities, youth from Quetta too considered the government policies (t = 14.555; *p* = 0.000), lack of youth participation (t = 11.253; *p* = 0.001), employment opportunities (t = 13.443; *p* = 0.000), and social engagement (t = 10.021; *p* = 0.001) as important factors that impact youth empowerment and entrepreneurship. The results are presented in Table 7.

**Table 7:** Regression analysis (Quetta).

**Sr. No**	**Relationship**	**T-Value**	***P*** **-Value**	**Decision**
1.	Government policies → youth empowerment and entrepreneurial initiatives	14.555	0.000	Accepted
2.	Lack of youth participation → youth empowerment and entrepreneurial initiatives	11.253	0.001	Accepted
3.	Employment opportunities → youth empowerment and entrepreneurial initiatives	13.443	0.000	Accepted
4.	Social engagement → youth empowerment and entrepreneurial initiatives	10.021	0.001	Accepted

GP, Government policies; LYP, Lack of youth participation; EO, Employment opportunities; SE, Social engagement; YEI, Youth empowerment and employment initiatives.

## Discussion

Against the backdrop of government policies and their impact on youth empowerment and entrepreneurial initiatives, research was conducted in different cities across the country and interesting findings have been documented. It is evident from the results that the respondents, drawn from different parts of the country, are of the view that government policies have a significant positive impact on the motivation of youngsters to embark on entrepreneurial initiatives and empower themselves. Similar findings have been reported by Augsberger et al. (2019) in the US, Ogamba (2019) in Nigeria, and (Geza et al., 2022) in South Africa. This reinforces our finding that policies devised by the government can either empower youth or suppress their entrepreneurial motivations.

On the impact of political participation by youth on their empowerment and entrepreneurial initiatives, respondents from the provincial and federal capital cities of Pakistan agreed that governments tend to ignore the participation of youth in political processes and policy development procedures. Youth are the biggest stakeholders as the future of the nation rests in their hands. The political system also makes it difficult for the youth to participate in it. The same strained relationship has been studied by others (Taufiq et al., 2019) in rural areas of Pakistan, (Kitanova, 2020) in the EU, and (Weiss, 2020) in Germany where youth do not have enough opportunities to participate in the political process of the country. This further strengthens the results of this study and implies that more participation by young people in politics can empower them and encourage them to initiate entrepreneurial ventures.

The next hypothesized relationship is the impact of employment opportunities on youth empowerment. The results collected from the respondents show that the presence or absence of job opportunities in society is directly proportional to the entrepreneurial initiatives taken by the youngsters and their level of empowerment. In Pakistan, there is a lack of employment opportunities which further demotivates the youngsters from engaging in any entrepreneurial activity. The impact of employment opportunities has been examined by scholars in other contexts. For instance, Sumberg et al. (2021) studied the employment crisis among youth in Africa, Uju and Racheal (2018) examined the unemployment issue in Nigeria, and Abdullah and Othman (2021) studied the youth employment problem in the Kurdistan region and all of them came to the same conclusion that unemployment has a significant impact on youth empowerment and their entrepreneurial initiatives.

Finally, on the hypothesized relationship between youth social engagement and youth empowerment and entrepreneurship, this study found a positive impact. The results demonstrate that a high level of social engagement can motivate youth to undertake entrepreneurial initiatives and empower themselves. Social engagement has been examined by other scholars such as Huda et al. (2019), Dahles et al. (2020); and Sirine et al. (2020) and reached similar conclusions as this study.

Table 8 provides a comparative overview of the cities from where the data was collected. The analysis shows that all the hypothesized factors were considered important factors that affect youth empowerment and employment initiatives. The policies devised by the government emerged as a crucial factor that impacts the youth. In all the major cities of the country, the youth responded that the policies directed toward the youth are not effective because they were not involved or consulted while formulating policies for their future. Moreover, these policies have never been implemented in true letter and spirit so that young people could benefit from them. The analysis also showed that respondents from Islamabad, Lahore, Peshawar, and Quetta consider that in addition to ineffective and inadequate youth-related government policies, youth themselves seem unmotivated to take any self-initiative. However, youth from Karachi did not conform to this notion. The reason could be that youth have never been mobilized in a manner that motivated them to create their own employment opportunity.

**Table 8:** Comparative analysis of the results.

	**Islamabad**		**Karachi**		**Lahore**		**Peshawar**		**Quetta**	
**Relationship**	**T-Value**	***P*** **-Value**	**T-Value**	***P*** **-Value**	**T-Value**	***P*** **-Value**	**T-Value**	***P*** **-Value**	**T-Value**	***P*** **-Value**
GP → YEI	12.104	0.000	14.113	0.000	13.227	0.000	10.227	0.001	14.555	0.000
LYP → YEI	24.841	0.000	3.255	0.013	12.255	0.000	12.674	0.000	11.253	0.001
EO → YEI	12.161	0.000	13.223	0.000	13.223	0.000	11.484	0.001	13.443	0.000
SE → YEO	4.581	0.000	3.001	0.012	11.001	0.001	12.001	0.000	10.021	0.001

GP, Government policies; LYP, Lack of youth participation; EO, Employment opportunities; SE, Social engagement; YEI, Youth empowerment and employment initiatives.

It was also evident that employment opportunities are lacking in the country. The number of graduating students is increasing per year whereas the government has failed to create opportunities and employment for all of them. All the respondents from different cities unanimously agreed that employment opportunities were rare and this significantly impacted youth empowerment. It demotivated the youth and made them feel less empowered. The general sense of disempowerment also had a negative impact on youth taking up entrepreneurial initiatives. The analysis further showed that youth engagement in social activities can boost their morale to feel empowered and engage in entrepreneurial activities. Prominent business houses and the private sector should organize such activities to engage the youth in their community and motivate them to engage in entrepreneurial activities. However, the respondents from Karachi considered empowerment and initiation of entrepreneurial activities as internal motivation factors of an individual.

This study established that youth empowerment and entrepreneurship are affected by all the hypothesized factors. The policies of the government, the reluctance of the youth to take initiative, scarce employment opportunities, and the lack of social engagement among youngsters prevent them from having a broader vision and inhibit their entrepreneurial activities.

## Conclusion

There are various studies worldwide on youth development, however, very few focus on youth empowerment and entrepreneurial initiatives within a framework of youth development. This research gap was particularly evident in Pakistan, a developing country. Entrepreneurial activities are the economic backbone of any country, and it serves as the cornerstone for numerous job creation and youth empowerment in society. The Pakistani institutes of higher education tend to churn out many graduates into a job market where employment opportunities are minimal which further makes it imperative to empower and motivate youth to be entrepreneurial. The factors that were identified to have an impact on the entrepreneurial behavior of young Pakistanis have been tested in the form of hypotheses and regression analysis was used for the analysis. There is a significant mismatch between the huge number of employable youth and the corresponding availability of resources to help them with good employment. Governmental policies have been questioned at every stage for not catering to the needs of young people. To empower the youth the government needs to develop such strategies which would lead to mainstreaming the youth in developmental planning actions, especially directed toward the empowerment of youngsters and assessment of the consequences of the developmental activities.

### Theoretical implications

This study presented a framework of factors that contributes to the existing literature on youth empowerment and entrepreneurship in Pakistan. The perspectives of youth regarding factors such as government policies, lack of youth participation, and lack of employment opportunities have been collected from the responses to the questionnaire. The results of the study suggest that the empowerment of the youth at individual and collective levels would ultimately yield positive outcomes.

### Social implications

The government can undertake a need analysis to realistically profile the developmental situation around the country. The development of a youth policy aimed at the engagement of youth should be based on a real analysis of the situations of youngsters. Youth from minority groups should be included in the youth empowerment policies on a special basis so that everyone feels included in the national policy. Furthermore, to make the country prosperous, youth participation is fundamental. Their participation can be encouraged by designing and implementing youth programs in letter and spirit. The participation of youngsters can also be increased by educating them and initiating capacity-building programs. Youth awareness can also be heightened by initiating campaigns using modern means such as the internet, text messages, and social media platforms.

There are several reasons behind youth unemployment in Pakistan such as financial crisis, unfocused government policies, and economic conditions of the country. The findings of this study implicate the lack of employment opportunities, but the provision of the right opportunities for entrepreneurship can motivate and empower young entrepreneurs. The policy makers need to realize that participation of youth while formulating national policies is the only solution to the issues being faced by young people. The youth are not just the future but they are also the present in the context of Pakistan and must be prioritized accordingly in every field.

### Limitations and future recommendations

Future research can adopt a longitudinal research design to examine other conclusions. The model could be tested further with other factors and in different contexts to yield different results. The data could be collected from other respondents from different demographic profiles and compare the findings with the results from this study on youngsters. Comparison between countries can also reveal interesting insights on youth perspectives from other countries.

## Data availability statement

The raw data supporting the conclusions of this article will be made available by the authors, without undue reservation.

## Ethics statement

The studies involving human participants were reviewed and approved by Ethical Committee University of Wah. Written informed consent for participation was not required for this study in accordance with the national legislation and the institutional requirements.

## Author contributions

AJ: manuscript writing and data analysis. ZM: data gathering. MYK: literature review. MA: analysis and interpretation. SM and SSW: draft revision. All authors contributed to the article and approved the submitted version.

## Conflict of interest

The authors declare that the research was conducted in the absence of any commercial or financial relationships that could be construed as a potential conflict of interest.

## Publisher's note

All claims expressed in this article are solely those of the authors and do not necessarily represent those of their affiliated organizations, or those of the publisher, the editors and the reviewers. Any product that may be evaluated in this article, or claim that may be made by its manufacturer, is not guaranteed or endorsed by the publisher.
